# Global Gene Networks in 3D4/31 Porcine Alveolar Macrophages Treated with Antigenic Epitopes of *Actinobacillus pleuropneumoniae* ApxIA, IIA, and IVA

**DOI:** 10.1038/s41598-019-41748-3

**Published:** 2019-03-27

**Authors:** Suji Kim, Myung Whan Oh, Woo Bin Park, Han Sang Yoo

**Affiliations:** Department of Infectious Diseases, College of Veterinary Medicine, Seoul National University, Seoul, Republic of Korea

## Abstract

*Actinobacillus pleuropneumoniae* (App) is the causative agent of porcine pleuropneumonia. Although App produces several virulence factors, Apx toxins, the primary App virulence factors, have been the focus of numerous studies. However, the host response against the Apx toxins has not been elucidated at the transcriptomic level. Therefore, in this study, we examined the response of an immortalized porcine alveolar macrophage cell line (IPAM 3D4/31) to four antigenic epitopes of the App exotoxins, ApxIA, IIA and IVA. The antigenic epitopes of the Apx toxins (ApxIA Ct, ApxIIA Nt, ApxIVA C1 and ApxIV C2) were determined by an *in-silico* antigenicity prediction analysis. Gene expression in IPAMs was analyzed by RNA-Seq after treatment with the four proteins for 24 h. A total of 15,269 DEGs were observed to be associated with cellular and metabolic processes in the GO category Biological Process and nuclear receptors and apoptosis signaling in IPA analyses. These DEGs were also related to M2 macrophage polarization and apoptosis in IPAMs. These host transcriptional analyses present novel global gene networks of the host response to treatment with Apx toxins.

## Introduction

*Actinobacillus pleuropneumoniae* cytolysin (Apx toxin) is an important virulence factor of *A. pleuropneumoniae* (App), which is a causative agent of porcine pleuropneumoniae (PP). Apx is a pore-forming toxin belonging to the RTX (Repeats in ToXin) family of toxins that are produced by several bacterial pathogens, including *Pasteurellaceae*^1^. RTX toxins damage the membrane of the target cell and ultimately cause cell lysis^2,3^. However, the interaction of Apx toxins with target cells is poorly understood, because of the complex nature of the production of this exotoxin by App^4^. In particular, research on the interaction between ApxIV and host cells is difficult since it is only expressed *in vivo*^2,5–8^.

Alveolar macrophages play a critical role in the evasion of host defenses after infection. Apx toxins impair the phagocytic and chemotactic function of macrophages to avoid host clearance^9^. The ApxI, ApxII, and ApxIII toxins are fatal to neutrophils and macrophages^10–12^, whereas the ApxIV toxin may damage macrophages and PMNs, but this activity has not yet been determined^13^. The ApxI toxin leads to the apoptosis of alveolar macrophages by activating the p38 and JNK signaling pathways^14^. ApxI-induced apoptosis can reduce bactericidal activity, and activate the caspase-8-Bid-caspase-9 pathways. The activation of both the caspase-8- and caspase-9-mediated apoptotic pathways induces the phosphorylation of the p38 and JNK MAPKs, leading to ApxI- induced apoptosis in PAMs via the MAPK- and caspase-dependent pathways^15^. RTX toxins cause cell lysis and necrosis at high concentrations and apoptosis via signaling cascades at low concentrations^16–21^.

The two primary types of polarized macrophages are M1 and M2 macrophages. A classical phenotype of M1 macrophages is the production of pro-inflammatory cytokines, whereas that of M2 macrophages is the production of anti-inflammatory factors^22–24^. Peroxisome proliferator-activated receptors (PPARs) are ligand-dependent nuclear transcription factors that significantly enhance anti-inflammatory effects. One PPAR isoform, PPARγ, exerts anti-inflammatory activity in macrophages to regulates the immune inflammatory response^25,26^. Activation of the PPARγ pathway is important for regulating alternatively activated (M2) macrophages^27,28^. Subsequent studies have reported that the absence of PPARγ expression leads to compromised M2-type responses^27^ and can negatively control the cell cycle in relation to lung pathogenesis^29^.

Transcriptomic analyses of App and host gene expression have been conducted in several studies. An analysis of App gene expression in infected lungs was carried out to identify App survival mechanisms used to counteract hostile host responses^30,31^. Host transcriptional analyses of App-infected porcine lung have also been performed^32,33^, and hilar lymph node and liver analyses were carried out by microarray^34,35^. Recently, changes in gene expression during the App-host interaction were reported^36,37^. These studies compared the expression of genes in relation to pathogen invasion mechanisms and the host immune response. In addition, many studies have been conducted to identify the changes in gene expression in the host and App. However, a transcriptomic analysis of Apx toxin-treated host cells has not yet been performed.

Therefore, in this study, the transcriptional responses of an immortalized porcine alveolar macrophage cell line (IPAM 3D4/31) treated with antigenic epitopes of ApxIA, ApxIIA, and ApxIVA were analyzed to identify the mechanism by which Apx toxins affect host cells. We evaluated the host response by identifying differentially expressed genes (DEGs) of IPAMs treated with ApxA toxins. The results of this study will be foundational to our understanding of the host response to Apx toxins and aid in the development of control measures against App infection.

## Results

### Preparation of recombinant antigenic epitopes of ApxIA, IIA, and IVA

Recombinant antigenic epitopes of ApxIA Ct, ApxIIA Nt, ApxIVA C1 and ApxIVA C2 were designed using a multiple sequence alignment and were selected based on a low percent identity and antigenicity prediction analysis. The sequences encoding antigenic epitopes of the Apx toxins were cloned into pET vectors, and their expression was induced in *E. coli* with IPTG. The expressed proteins were analyzed by SDS-PAGE and Western blot after purification via nickel-nitrilotriacetic acid (Ni-NTA) chelate affinity chromatography. An SDS-PAGE analysis showed that the molecular masses of the recombinant ApxIA Ct, ApxIIA Nt, ApxIVA C1, and ApxIVA C2 proteins were approximately 24.2, 19.1, 55, and 46.2 kDa, respectively. The purified recombinant proteins were confirmed by Western blot with an anti-His antibody (Supplementary Fig. 1).

### Mapping and annotation of RNA-Seq data

The transcriptomes of IPAMs treated with the four recombinant proteins Apx toxin epitopes for 24 h were analyzed by RNA-Seq. Ten cDNA libraries from the cells treated with ApxIA Ct, ApxIIA Nt, ApxIVA C1, ApxIVA C2 and DPBS were sequenced. After quality filtering, the RNA-Seq assay yielded 617 million 101 bp paired-end clean reads, varying from 28 to 32 million for each sample. Among the clean reads, approximately 95.24% uniquely mapped onto version 11.1 of the *Sus scrofa* genome. Gene expression was quantified by converting the raw reads to FPKM. A total of 15,269 genes (mean FPKM of the 10 samples >0) were expressed in IPAMs with 30,511 gene annotations in version 11.1 of the *S. scrofa* genome.

### Differentially expressed genes in the Apx toxin-treated IPAMs

**Figure 1:**
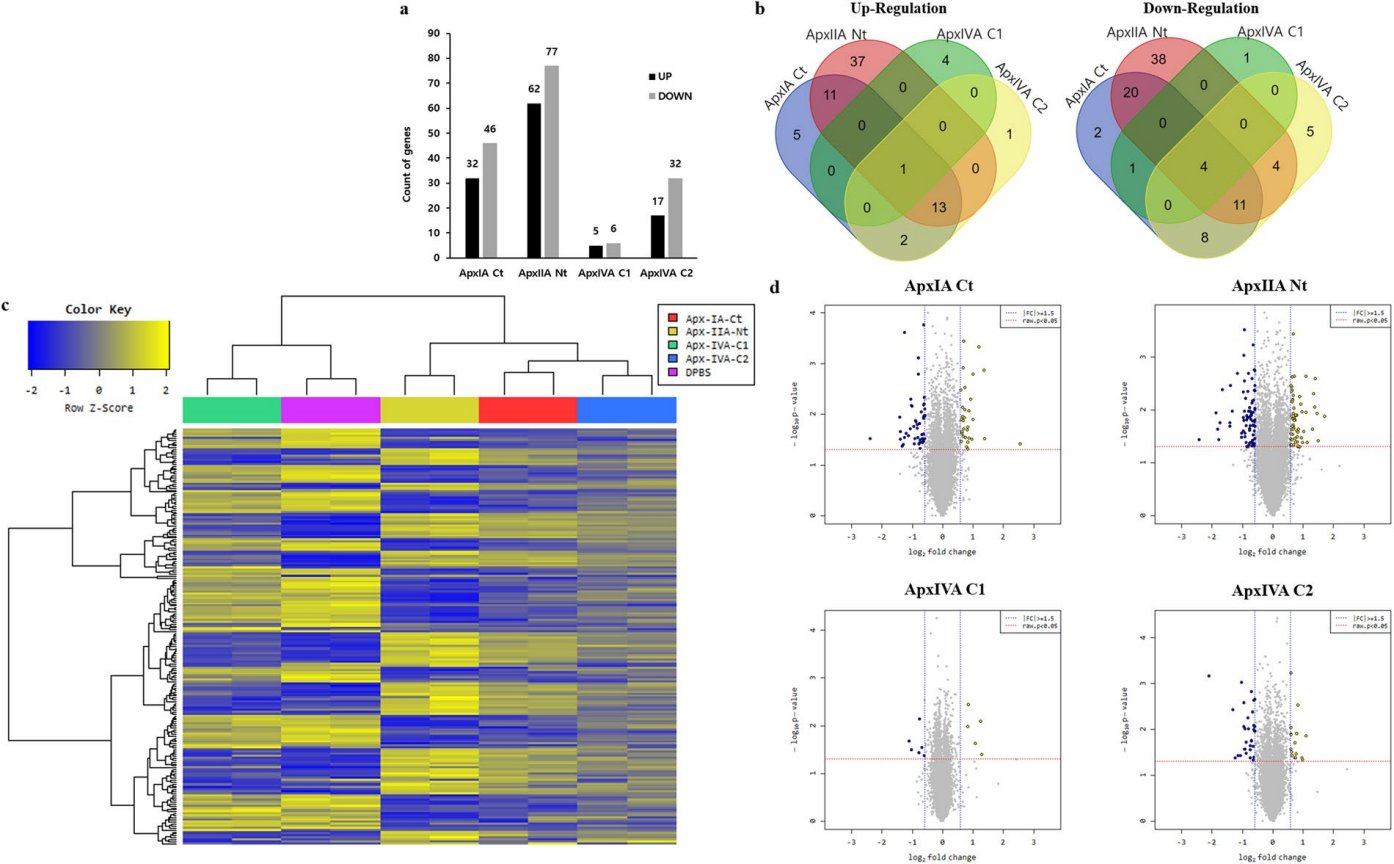
Gene expression levels of IPAMs treated with Apx toxins: ApxIA Ct, ApxIIA Nt, ApxIVA C1, and ApxIVA C2. (**a**) The number of significantly up and down regulated genes, (**b**) Venn diagram analysis, (**c**) Clustering analysis, and (**d**) Volcano plot analysis in IPAMs treated with Apx toxins. The blue dotted line indicates an expression level of |fold change| ≥ 1.5. The red dotted line indicates an expression level with a *p*-value < 0.05. The expression levels were calculated using the base-2 logarithm of the normalized hybridization signals from each sample.

In our analysis, 208 DEGs (|fold change| ≥ 1.5, *p*-value < 0.05) were detected, in Apx toxin-treated IPAMs compared with those treated with DPBS. Of these, 78, 139, 11 and 43 were found in IPAMs treated with ApxIA Ct, ApxIIA Nt, ApxIVA C1 and ApxIVA C2, respectively (Fig. 1a). In most cases, the upregulation of one gene (HMGCS1) and the downregulation of four genes (ABCA1, ABCG1, MGP, and NTS) were observed in the Apx toxin-treated IPAMs (Fig. 1b). Hierarchical clustering was divided into two groups: one from IPAMs treated with ApxIA Ct, ApxIIA Nt, and ApxIVA C2 and the other from IPAMs treated with ApxIVA C1 and DPBS (Fig. 1c). In a Volcano plot analysis of the DEGs from the Apx toxin-treated IPAMs, the highest and lowest expression levels were observed for cells treated with ApxIIA Nt and ApxIVA C1, respectively (Fig. 1d). The raw files and normalized datasets are available at Gene Expression Omnibus (GEO) (http://www.ncbi.nlm.nih.gov/geo website) under accession number GSE116263.

### Functional annotation of differentially expressed genes in Apx toxins-affected IPAMs

**Figure 2:**
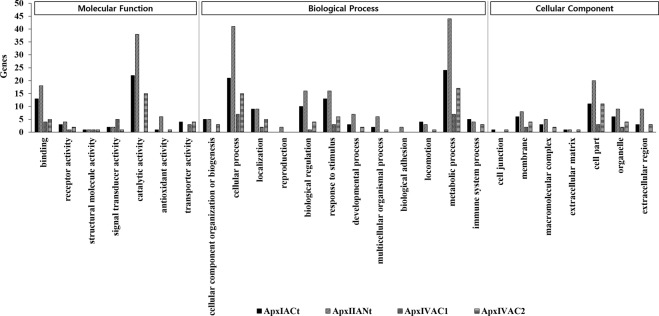
Gene ontology (GO) analyses of annotated genes in IPAMs treated with Apx toxins. The annotated genes were divided into the main GO-terms biological processes, molecular function, and cellular components and further divided into subcategories.

A gene set enrichment analysis of GO terms was carried out based on the 55, 98, 11, and 36 DEGs in IPAMs, treated with ApxIA Ct, ApxIIA Nt, ApxIVA C1 and ApxIVA C2, respectively. PANTHER annotation revealed that the highest DEGs were observed in cells treated with ApxIIA Nt, followed by ApxIA Ct, ApxIVA C2, and ApxIVA C1. Through PANTHER classification, genes related to catalytic activity in Molecular Function, cellular and metabolic processes in Biological Process and cell part in Cellular Components had the highest DEGs. The ratios of DEGs in Molecular Function were 47.8%, 55.1%, 35.7% and 51.7% in the “catalytic activity” category in IPAMs treated with ApxIA Ct, ApxIIA Nt, ApxIVA C1 and ApxIVA C2, respectively. The highest numbers of DEGs from IPAMs treated with ApxIA Ct, ApxIIA Nt, ApxIVA C1 and ApxIVA C2 in the Biological Process category were associated with “cellular process” (21.9%, 26.5%, 35.0% and 26.3%, respectively) and “metabolic process” (25.0%, 28.4%, 35.0% and 29.8%, respectively). The most abundant DEGs in the Cellular Component category were associated with “cell part”, which accounted for 35.5%, 38.5%, 42.9%, and 42.3% of IPAMs treated with ApxIA Ct, ApxIIA Nt, ApxIVA C1 and ApxIVA C2, respectively (Fig. 2).

### Canonical pathway analysis

Of the 4,621 DEGs that mapped to the Ingenuity Knowledge Base and passed the dataset filter (*p*-value), 1,286, 1,523, 658 and 911 DEGs of IPAMs treated with ApxIA Ct, ApxIIA Nt, ApxIVA C1, and ApxIVA C2, respectively, were analyzed. These DEGs were used in a core analysis carried out by using Ingenuity^®^ Pathways Analysis (IPA, Ingenuity Systems, www.ingenuity.com). Canonical pathways were identified for the differentially expressed genes of IPAMs treated with ApxIA Ct, ApxIIA Nt, ApxIVA C1, and ApxIVA C2. Forty different canonical pathways in IPAMs treated with ApxIA Ct, ApxIIA Nt, ApxIVA C1, and ApxIVA C2 were identified with significant differences [(−log(*p*-value) ${\rm{\ge }}1.3$] (Supplementary Tables 1–4). Metabolic pathways accounted for only 5, 7, 9, and 3 of the 40 significant canonical pathways of DEGs in IPAMs treated with ApxIA Ct, ApxIIA Nt, ApxIVA C1 and ApxIVA C2, respectively. Among the significant canonical pathways of DEGs in ApxIA Ct-treated IPAMs, apoptosis signaling, TNFR1 signaling and death receptor signaling were related to “Apoptosis”, while glucocorticoid receptor signaling, integrin signaling and PI3K/AKT signaling were involved in “Intracellular and Second Messenger Signaling”. The IPAMs treated with ApxIIA Nt also had DEGs associated with several signaling pathways including “Intracellular and Second Messenger Signaling” (sirtuin signaling pathway, insulin receptor signaling, and the unfolded protein response), and “Cellular Stress and Injury” (role of BRCA1 in DNA damage response, NRF2-mediated oxidative stress response, and ATM signaling). Significant canonical pathways in ApxIVA C1-treated cells were enriched in phospholipase C signaling, protein kinase A signaling, and the sirtuin signaling pathway in “Intracellular and Second Messenger Signaling” and for iNOS signaling, PI3K signaling in B lymphocytes, and p38 MAPK signaling in “Cellular Immune Response”. The canonical pathways in ApxIVA C2-treated IPAMs were primarily associated with actin nucleation by the ARP-WASP complex, integrin signaling and glucocorticoid receptor signaling in “Intracellular and Second Messenger Signaling” and granulocyte adhesion and diapedesis, granulocyte adhesion and diapedesis, and IL-10 signaling in “Cellular Immune Response”.

### Analysis of signaling pathways in the Apx toxins-treated IPAMs

**Figure 3:**
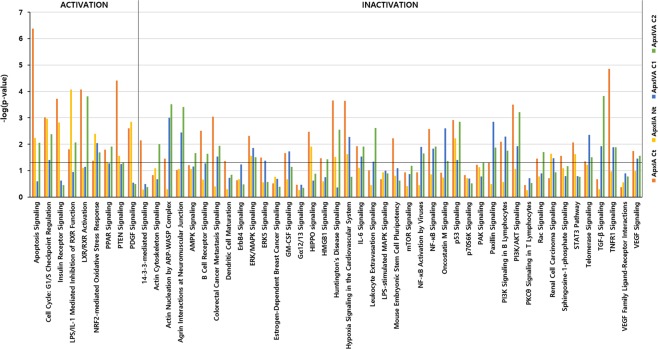
Signaling pathways of Apx toxins-treated IPAMs. The names of the signaling pathways are shown on the y-axis. The − log of the *p*-value calculated using Fisher’s exact test is shown on the top x-axis. The signaling pathway analyses were generated through the use of IPA (QIAGEN Inc., https://www.qiagenbioinformatics.com/products/ingenuity-pathway-analysis)^75^.

According to the signaling pathway classification, the DEGs of IPAMs were commonly enriched in the “Apoptosis”, “Nuclear Receptor Signaling”, “Cellular Immune Response” and “Humoral Immune Response” categories. Based on the z-scores, apoptosis signaling, PTEN signaling involved in “Apoptosis” and LXR/RXR activation, PPAR signaling related to “Nuclear Receptor Signaling” were commonly predicted to be activated, while B cell receptor signaling and HMGB1 signaling involved in the “Humoral Immune Response” and NF-κB signaling and IL-6 signaling related to the “Cellular Immune Response” were commonly predicted to be inactivated (Fig. 3).

Among the signaling pathways of IPAMs treated with ApxIA Ct, ApxIIA Nt and ApxIVA C2, macropinocytosis signaling, MIF regulation of innate immunity and CD40 signaling related to the “Cellular Immune Response” were commonly expressed. However, micropinocytosis signaling and MIF regulation of innate immunity were commonly predicted to be inhibited, although CD40 signaling was predicted to be activated only in the ApxIVA C2-treated cells according to the z-score. The DEGs also significantly mapped to the signaling pathways related to “Apoptosis”, comprising toll-like receptors signaling, induction of apoptosis by H1V1 and TWEAK signaling. Induction of apoptosis by H1V1 was commonly predicted to be activated in the ApxIA Ct-, ApxIIA Nt- and ApxIVA C2-treated cells.

### Signaling pathways of Apx toxins-treated IPAMs associated with M2 macrophage polarization

**Figure 4:**
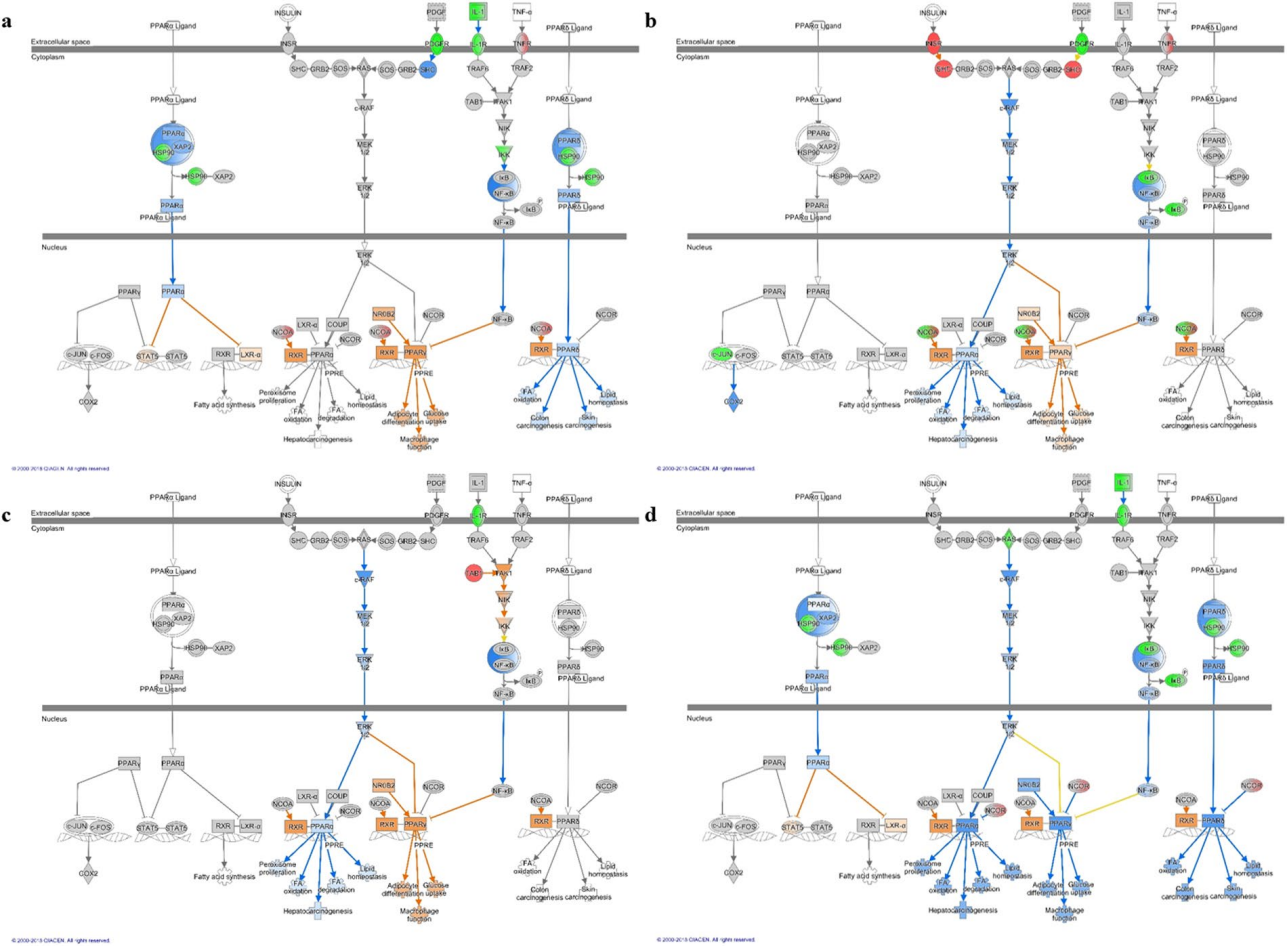
Ingenuity pathway analyses of the PPAR signaling pathway in IPAMs treated with Apx toxins. (**a**) ApxIA Ct, (**b**) ApxIIA Nt, (**c**) ApxIVA C1, and (**d**) ApxIVA C2. Genes shown in red indicates up-regulation, green indicates down-regulation, orange indicates predicted activation, blue indicates predicted inhibition, and an uncolored node indicates that the genes were not differentially expressed in this pathway. The Ingenuity pathway analyses were generated through the use of IPA (QIAGEN Inc., https://www.qiagenbioinformatics.com/products/ingenuity-pathway-analysis)^75^.

An analysis of significant signaling pathways associated with macrophage polarization was carried out (Table 1). The results indicated that glucocorticoid receptor signaling, GM-CSF signaling, IL-10 signaling, IL-6 signaling, LXR/RXR activation, NF-κB activation by viruses, NF-κB signaling, PI3K signaling in B lymphocytes, PI3K/AKT signaling and PPAR signaling were commonly expressed and showed consistent activation states in cells treated with ApxIA Ct, ApxIIA Nt, ApxIVA C1 and ApxIVA C2. Among these pathways, LXR/RXR activation and PPAR signaling associated with M2 polarization were commonly activated. However, GM-CSF signaling, IL-6 signaling, NF-κB activation by viruses and NF-κB signaling associated with M1 polarization were suppressed or exhibited a z-score of 0, in the IPAMs treated with ApxIA Ct, ApxIIA Nt, ApxIVA C1 and ApxIVA C2. In particular, NF-κB, IL-1, and ERK were down regulated in the signaling pathways of IPAMs treated with the four Apx recombinant proteins. These molecules can induce the activation of PPARγ, which results in M2 differentiation. PPARγ was commonly predicted to be activated according to the observed z-score for PPAR signaling in ApxIA Ct-, ApxIIA Nt- and ApxIVA C1-treated cells. However, PPARγ was predicted to be inhibited in the ApxIVA C2 treatment group, because NCOR was predicted to be activated based on the z-score (Fig. 4). The real-time PCR results showed that M1 macrophage activation-related genes were not significantly expressed at 12 h after infection, while M2 macrophage activation-related genes were significantly up regulated after 24 h of infection in four Apx toxins-treated IPAMs. Although *GM-CSF*, *IL6* and *IL12p35* were significantly up regulated after 12 h of infection, they were not up regulated after 24 h of infection, and additionally, *TNF-α* was not significantly expressed in IPAMs. On the other hand, M2 macrophage-related genes (*PPARγ*, *TGF-β*, *STAT3*, *IL5* and *STAT6*) were significantly up regulated after 24 h of infection (Supplementary Fig. 3).

**Tablee 1:** Canonical pathways associated with macrophage polarization of IPAMs treated with Apx toxin.

Ingenuity Canonical Pathway	ApxIA Ct			ApxIIA Nt			ApxIVA C1			ApxIVAC2		
	−log(p-value)	Ratio	z-score	−log(p-value)	Ratio	z-score	−log(p-value)	Ratio	z-score	−log(p-value)	Ratio	z-score
Glucocorticoid Receptor Signaling	4.32	0.11	—	0.843	0.0812	—	1.7	0.0493	—	2.59	0.0725	—
GM-CSF Signaling	1.67	0.122	−0.333	0.676	0.0946	−0.378	1.73	0.0811	−1.342	1.15	0.0811	0
IL-1 Signaling	0.8	0.0842	−1.134				1.74	0.0737	0.378	2.38	0.105	−0.378
IL-10 Signaling	1.31	0.11	—	0.461	0.0822	—	1.76	0.0822	—	2.66	0.123	—
IL-15 Production	0.331	0.0714	—				1.36	0.107	—			
IL-6 Signaling	1.93	0.109	−1.069	1.11	0.102	0	1.53	0.0625	−1.414	1.91	0.0859	−0.632
iNOS Signaling	1.31	0.125	−0.447	1.92	0.167	0.816	2.65	0.125	0.447	1.4	0.104	0.447
JAK/Stat Signaling	2.21	0.133	−0.302	0.752	0.0964	−0.707	1.06	0.0602	−0.447	0.643	0.0602	0.447
LPS/IL-1 Mediated Inhibition of RXR Function	1.81	0.0925	1.134	4.07	0.137	0.277	0.948	0.0441	1.342	2.07	0.0749	1.667
LXR/RXR Activation	4.07	0.148	2.324	1.11	0.102	0.707	1.14	0.0547	1.633	3.81	0.117	1.508
NF-κB Activation by Viruses	0.935	0.0909	−2.121	0.449	0.0795	−0.378	1.9	0.0795	−1.89	1.65	0.0909	−1.414
NF-κB Signaling	2.57	0.11	−1.342	0.86	0.0884	0	1.84	0.0608	−0.302	1.91	0.0773	0
PI3K Signaling in B Lymphocytes	2.09	0.111	−0.832	0.565	0.0815	−0.632	2.29	0.0741	−1.897	1.76	0.0815	−1
PI3K/AKT Signaling	3.5	0.138	−1.698	1.07	0.1	−1.155	1.93	0.0692	−0.333	3.22	0.108	−2.111
PPAR Signaling	1.79	0.116	2.714	1.33	0.116	0.302	1.28	0.0632	1.633	1.91	0.0947	2.333
PPARα/RXRα Activation	1.82	0.0973	1.732	0.803	0.0865	−0.577	2.18	0.0649	0.333	2.57	0.0865	0.632
Role of JAK2 in Hormone-like Cytokine Signaling	1.99	0.176	—	1.16	0.147	—	0.205	0.0294	—			
STAT3 Pathway	2.07	0.133	−1.265	1.62	0.133	−0.632	0.787	0.0533	−1	0.764	0.0667	−0.447
TGF-β Signaling	0.679	0.0805	−0.816	0.292	0.069	−2	1.93	0.0805	0	3.83	0.138	−1.265
TNFR1 Signaling	4.85	0.24	−0.302	0.969	0.12	−0.816	1.88	0.1	−1.342	1.88	0.12	−1.342
TNFR2 Signaling	2.26	0.2	−0.447	0.503	0.1	—	0.681	0.0667	—	0.95	0.1	—
Toll-like Receptor Signaling	0.888	0.0921	0	0.249	0.0658	0.447	1.19	0.0658	—	2.54	0.118	0.333
TREM1 Signaling	1.61	0.118	−0.333				0.437	0.0395	—	1.11	0.0789	0
VEGF Signaling	1.74	0.11	−0.333	0.993	0.101	−0.632	1.45	0.0642	−2.646	1.56	0.0826	−0.816

### Activation of apoptosis signaling in IPAMs treated with ApxIA Ct, ApxIIA Nt, and ApxIVA C2

**Figure 5:**
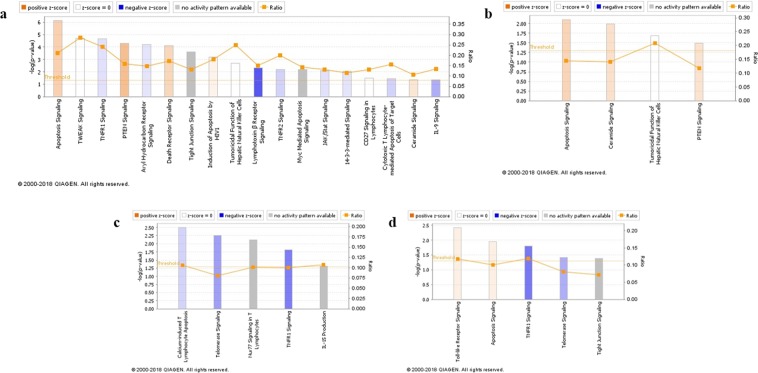
Canonical pathway analyses associated with apoptosis. (**a**) ApxIA Ct, (**b**) ApxIIA Nt, (**c**) ApxIVA C1, (**d**) ApxIVA C2. The −log of the *p*-value calculated using Fisher’s exact test is shown on the top y-axis. The ratio (orange dots) represents the number of genes in each dataset divided by the total number of genes in a given pathway. The colored bar-chart indicates the significance of the z-score and the directional effect of gene enrichment for each pathway. The canonical pathway analyses were generated through the use of IPA (QIAGEN Inc., https://www.qiagenbioinformatics.com/products/ingenuity-pathway-analysis)^75^.

An analysis of signaling pathways involved in apoptosis was performed (Fig. 5). In total, 18, 4, 5, and 5 significant pathways [(−log(*p*-value) ${\rm{\ge }}1.3$] associated with apoptosis were observed in IPAMs treated with ApxIA Ct, ApxIIA Nt, ApxIVA C1, and ApxIVA C2, respectively. Among the signaling pathways of ApxIA Ct-treated cells, apoptosis signaling, PTEN signaling, death receptor signaling, induction of apoptosis by H1V1, and ceramide signaling were predicted to be activated. Of the signaling pathways expressed in ApxIIA Nt-treated cells, apoptosis signaling, ceramide signaling, and PTEN signaling were also predicted to be activated. However, ApxIVA C1 did not predict any predicted pathways associated with “Apoptosis”. Among the five signaling pathways in ApxIVA C2-treated cells related to “Apoptosis”, toll-like receptor signaling and apoptosis signaling were predicted to be activated. In particular, apoptosis signaling was commonly predicted to be activated and was more significant [−log(*p*-value) of 6.38, 2.24 and 2.05] than other significant pathways in the IPAMs treated with ApxIA Ct, ApxIIA Nt, and ApxIVA C2, respectively.

**Figure 6:**
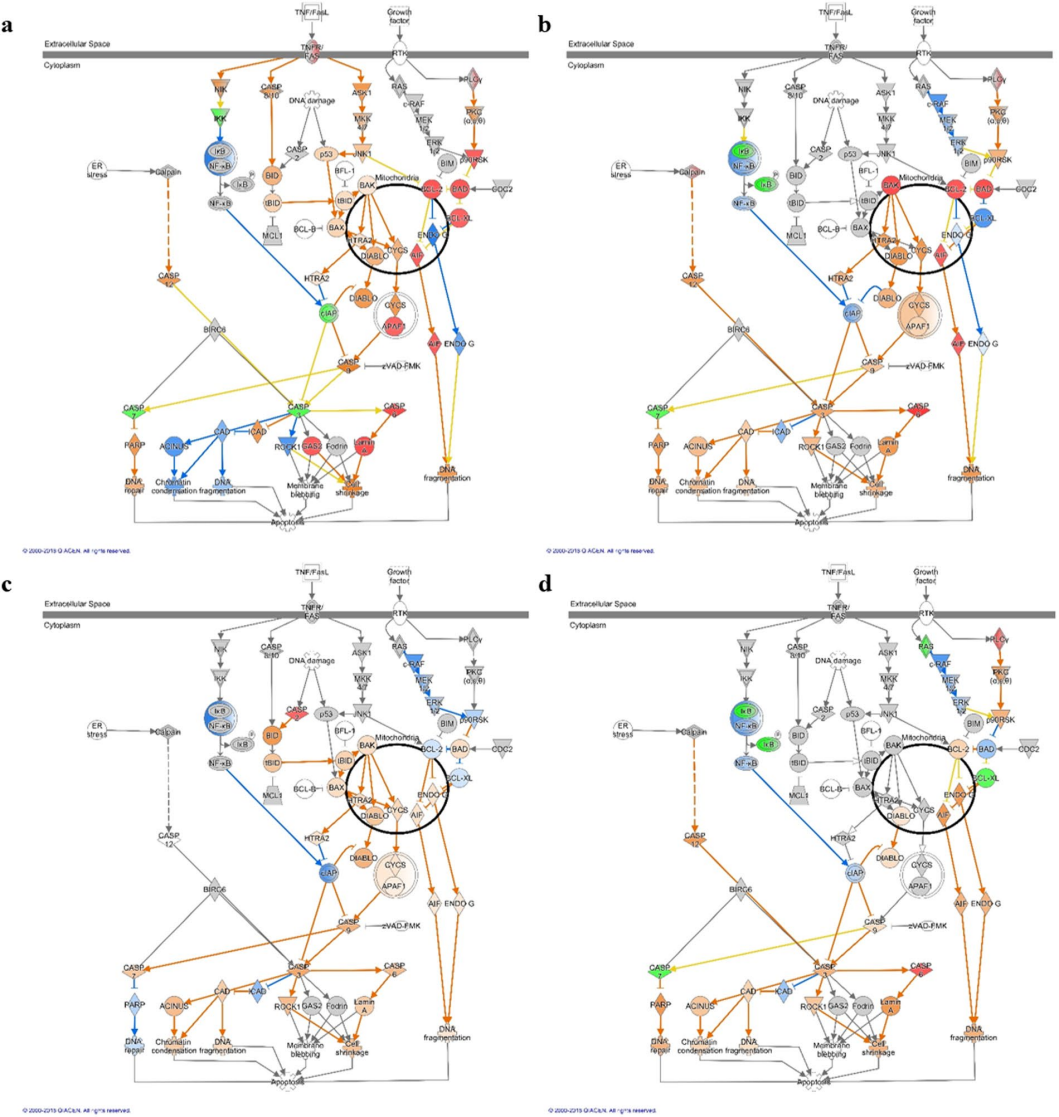
Ingenuity pathway analyses of apoptosis in IPAMs treated with Apx toxins. (**a**) ApxIA Ct, (**b**) ApxIIA Nt, (**c**) ApxIVA C1, (**d**) ApxIVA C2. The ingenuity pathway analyses were generated through the use of IPA (QIAGEN Inc., https://www.qiagenbioinformatics.com/products/ingenuity-pathway-analysis)^75^.

*Aif*, *Apaf1*, *Bad*, *Bak*, *Bcl2*, *Bcl-xl*, *Caspase 2*, *Caspase 3*, *Caspase 6*, *Caspase 7*, *Gas2*, *LaminA* and *p90rst* were the significantly expressed apoptosis signaling genes in ApxIA Ct-treated cells. The genes *Aif*, *Bad*, *Bak*, *Bcl2*, *Caspase 6* and *Caspase 7* were significantly expressed in the apoptosis signaling pathway of ApxIIA Nt-treated cells. *Bcl2*, *Caspase 6* and *Caspase 7* were significantly expressed in the apoptosis signaling pathway of ApxIVA C2-treated cells. No DEGs in the apoptosis signaling pathway of ApxIVA C1-treated cells were significantly expressed. Among the genes expressed in the apoptosis signaling pathway of ApxIA Ct-, ApxIIA Nt- and ApxIVA C2-treated cells, *Caspase 6* was commonly up-regulated and *Caspase 7* was commonly down-regulated. The genes *Bak*, *Cycs*, *Apaf1*, *Caspase 9*, *Caspase 6* and *LaminA* were predicted to be activated in IPAMs treated with ApxIA Ct and ApxIIA Nt. The genes *Caspase 9*, *Caspase 3*, *Caspase 6* and *LaminA* were predicted to be activated in ApxIVA C2-treated cells (Fig. 6). *Apaf1*, *Bak*, *Caspase6*, and *Lamin A*, which are known as key factors of apoptosis, were identified as significant genes in this pathway. In the real-time PCR results, genes (*bak*, *bax*, *cytochrome c*, *apaf1*, *caspase 3*, *caspase 6* and *laminA*) related to apoptosis were significantly up regulated after 24 h of infection, except in ApxIVA C1-stimulated IPAMs (Supplementary Fig. 4).

### Validation of RNA-Seq by qRT-PCR

RNA samples were assayed by quantitative real-time RT-PCR (qRT-PCR) to validate the RNA-Seq results. The IPAMs treated with Apx toxins showed significant changes in gene expression compared to those in DPBS-treated cells. The up-regulated gene *hmgcs1*, the down regulated genes, *nts*, *mgp* and *abcg1* and the randomly expressed *vgf* gene were selected to assay by qRT-PCR using the same RNA samples as those used for the RNA-Seq analysis. The correlation coefficient between the two analyses was 0.906. Although the RNA-Seq data for some genes such as *abcg1* and *vgf* showed greater fluctuations (increase or decrease) compared to those of the qRT-PCR data, the differential expression of all selected genes was validated, as the qRT-PCR results showed the same trend, with respect to up-regulation or down-regulation (Supplementary Fig. 2).

## Discussion

Transcriptional studies of App-infected hosts have been conducted under *in vivo* and *in vitro* conditions to identify App infection mechanisms in the host^31,34,36,37^. However, a transcriptomic analysis to identify the effects of Apx toxins in the host had not been performed. Therefore, the goals of this study were to identify the invasive mechanisms of Apx toxins in an immortalized porcine alveolar macrophage cell line using partial epitopes of three types of Apx toxins. Transcriptomic analyses were performed using IPAMs treated with partial epitopes of ApxIA, ApxIIA, and ApxIVA. The results suggest possible roles for ApxIA Ct, ApxIIA Nt, and ApxIVA C2 in impairing the host defense system through the induction of apoptosis in IPAMs.

In this study, gene expression was analyzed in IPAMs treated with ApxIA Ct, ApxIIA Nt, ApxIVA C1, or ApxIVA C2. Among the identified signaling pathways, the DEGs of the ApxIA Ct-treated IPAMs were associated with “Apoptosis” and “Intracellular and Secondary Messenger Signaling”, those of ApxIIA Nt-treated cells were associated with “Intracellular and Second Messenger Signaling” and “Cellular Stress and Injury”, and those of ApxIVA C1- and ApxIVA C2-treated cells were enriched in “Intracellular and Second Messenger Signaling” and “Cellular Immune Response”. Signaling pathways of IPAMs treated with ApxIA Ct, ApxIIA Nt, ApxIVA C1, and ApxIVA C2 were commonly enriched in “Apoptosis”, “Nuclear Receptor Signaling”, “Humoral Immune Response” and “Cellular Immune Response”. Apoptosis signaling and PTEN signaling associated with “Apoptosis”, LPS/IL-1 mediated inhibition of RXR function, LXR/RXR activation and PPAR signaling associated with “Nuclear Receptor Signaling” were predicted to be activated, while B cell receptor signaling and HMGB1 signaling associated with “Humoral Immune Response”, GM-CSF signaling, IL-6 signaling, NF-κB signaling and PI3K/AKT signaling associated with “Cellular Immune Response” were predicted to be inhibited.

The activation states of macrophages are classified into classically activated (M1) and alternatively activated (M2), and these phenotypes are affected by different cytokines and microbial products^38^. M1 macrophages produce pro-inflammatory cytokines, such as TNF-α, IL-6, and IL-12. In contrast, M2 macrophages release anti-inflammatory cytokines, such as transforming growth factor (TGF)-β, IL-10, and IL-1 receptor antagonist^22–24^. In our study, polarization was predicted by the inhibition of pathways associated with M1 macrophage differentiation and the activation of pathways associated with M2 macrophage differentiation. In addition, nuclear receptor signaling was significantly enhanced in cells treated with the three Apx antigenic epitopes. The pathways associated with M1 macrophage polarization, including GM-CSF signaling, IL-6 signaling NF-κB activation by viruses and NF-κB signaling, were commonly predicted to be inhibited or had a z-score of 0 in the four antigenic epitope-treated IPAMs. However, the pathways associated with M2 macrophage polarization including LXR/RXR activation and PPAR signaling, were predicted to be activated. Of these signaling pathways, PPARγ was commonly predicted to be activated in the PPAR signaling pathway as a result of treatment with three Apx antigenic epitopes but not ApxIVA C2. In the PPAR signaling of ApxIVA C2-treated cells, PPARγ was predicted to be inhibited, because NCOR was predicted to be activated. However, inactivation of NF-κB and IL-1 can induce activation of PPARγ and these pathways were commonly down regulated with respect to PPAR signaling in IPAMs treated with the four antigenic epitopes. STATs, NF-κB and PPARs, known as leading transcription factors, drive a particular phenotype of macrophage polarization. Additionally, *PPARγ*, *TGF-β*, *STAT3*, *IL5* and *STAT6*, which are related to alternatively activated macrophages were significantly up-regulated after 24 h of stimulation with the four antigenic epitopes according to the quantitative real-time PCR results.

Nuclear receptors such as peroxisome proliferators activated receptor (PPAR) can induce the biological effects caused by toxins. PPARs function as important regulators of cell differentiation, proliferation, and apoptosis in macrophages. Importantly, of the various PPAR subtypes, PPARγ inhibits the expression of inflammatory cytokines and induces anti-inflammatory effects in the lung^39–42^. The importance of PPARγ in regulating the M2 phenotype of macrophages has been demonstrated by the fact that M2-type responses were compromised in the absence of PPARγ^26,27^. PPARγ expression is also important for the expression of characteristic genes in M2 macrophages^27,28^. These PPAR pathways induce anti-inflammatory responses to inhibit immune inflammatory genes (encoding IL-2, IL-6, IL-8, TNF-α, and metalloproteases) through the inhibition of NF-κB expression^43–48^. In particular, PPARγ can repress the activation of inflammatory response-associated genes by a SUMOylation-dependent pathway in macrophages, which induces the transrepression of NF-κB target genes^49^. The prediction of down-regulated NF-κB and up-regulated PPARγ in the PPAR pathway of IPAMs treated with the four Apx epitopes indicated that these pathways may be associated with M2 macrophage differentiation, and the inhibition of cellular immune responses in the analysis of signaling pathways also supports these results.

Among the signaling pathways observed to be associated through the Ingenuity Pathway Analysis, the apoptosis signaling pathway was significantly enriched in IPAMs treated with the ApxIA Ct, ApxIIA Nt, and ApxIVA C2 epitopes. However, all pathways associated with apoptosis were not enriched in ApxIVA C1-treated cells. The genes *caspase 9*, *caspase 6*, and *laminA* were commonly activated in the apoptosis signaling pathway in IPAMs treated with ApxIA Ct, ApxIIA Nt, and ApxIVA C2. In addition, the genes *bak*, *cycs*, and *apaf1* were activated in ApxIA Ct- and ApxIIA Nt-treated cells. We identified *apaf1*, *bak*, *caspase6*, and *laminA* as genes in the apoptosis pathway exhibiting significant differential expression in response to these Apx epitopes. Furthermore, *bak*, *bax*, *cytochrome c*, *apaf1*, *caspase3*, *caspase6* and *laminA* were significantly up-regulated after 24 h of stimulation with ApxIA Ct, ApxIIA Nt and ApxIVA C2.

Several studies have shown that toxins can kill target cells by inducing caspase activity^50–54^, in particular, toxins stimulate intracellular signals that induce apoptosis by intrinsic signaling pathways. Bak, a proapoptotic protein, is known as a crucial protein in the induction of intrinsic apoptosis^55^. Activation of bak results in mitochondrial membrane permeabilization^56,57^, which causes cytochrome c, a proapoptotic mitochondrial protein, to be translocated to the cytosol^58^. Cytochrome c induces the assembly of Apaf1 as well as procaspase-9, forming an “apoptosome”, which causes activation of the initiator caspase 9^59–61^. Activated caspase 9 causes DNA fragmentation and apoptosis through activation of caspase 3. Caspase 6 may be activated by the activation of caspase 3 and its activation is required for the formation of apoptotic bodies during apoptosis^62–66^. However, several studies showed that caspase 6 can activate procaspase 3, a downstream effector that causes activation of the cell death program^67,68^, and can act upstream of caspase 3^67^. In our study, caspase 6 was commonly activated in the apoptosis signaling of IPAMs treated with ApxIA Ct, ApxIIA Nt, and ApxIVA C2, while caspase 3 was only activated in ApxIIA Nt-, and ApxIVA C2-treated cells. However, Lamin A is activated by caspase 6, which eventually leads to apoptosis. Degradation of lamins, which are protease substrates, is mediated by the proteolysis of caspase 6. Lamins are a major component of the nuclear lamina and have nuclear functions during apoptosis^69,70^. In particular, *lamin A/C* plays an important role in nuclear apoptotic events such as in acting shrinkage, disassembly of the nuclear membrane and the formation of apoptotic bodies^62,63,71,72^. This analysis of signaling pathways showed that apoptosis through the intrinsic signaling pathway was commonly induced in IPAMs treated with ApxIA Ct, ApxIIA Nt, and ApxIVA C2. The genes *bak, bax, cytochrome C, apaf1, caspase3, caspase6* and *laminA* were also commonly activated in the apoptosis signaling pathway of ApxIA Ct-, ApxIIA Nt- and ApxIVA C2-treated cells.

In conclusion, the results of this study indicated that the antigenic epitopes of three types of Apx toxins showed different immune responses in an immortalized porcine alveolar macrophage cell line. The signaling pathways were enriched in the categories Apoptosis, Nuclear Receptor, Humoral Immune Response, and Cellular Immune Response. Of these pathways, activation of apoptosis, nuclear receptor signaling and inactivation of both humoral and cellular immune responses were commonly predicted. In particular, the pathways associated with M2 macrophage polarization were activated, including PPAR signaling pathways. Among the PPAR signaling pathways, PPARγ was activated in IPAMs treated with the four antigenic epitopes. In addition, activation of LXR/RXR and, PPAR signaling and inactivation of GM-CSF signaling, IL-6 signaling and NF-κB signaling were commonly predicted among the DEGs of IPAMs treated with the four Apx antigenic epitopes. This result indicates that the four antigenic epitopes of Apx toxins may induce M2 macrophage differentiation in IPAMs. The apoptosis signaling pathway was also commonly predicted to be activated in the IPAMs treated with ApxIA Ct, ApxIIA Nt, and ApxIVA C2. In particular, the Bak-APAF1-Caspase-9-Caspase-6-LaminA pathway was activated in the apoptosis of IPAMs induced by three Apx antigens. This result shows that ApxIA Ct, ApxIIA Nt and ApxIVA C2 may induce apoptosis in porcine alveolar macrophages and impair host defense systems. Future work will explore the role of the signaling pathways and the DEGs related to the pathogenesis of Apx exotoxin treatment to clarify the host response mechanism in porcine alveolar macrophages. This study will be helpful in understanding the host response to Apx toxins.

## Materials and Methods

### Cloning, expression and purification of antigenic epitopes of ApxIA, IIA and IVA

Partial epitopes of ApxA were designed based on low-percent identity and antigenicity prediction analyses used to select specific antigenic epitopes against the B-cell epitope in the encoded toxins ApxIA (AF363361.1), ApxIIA (AF3633362.1) and ApxIVA (HM021153.1). The C-terminal portion of ApxIA (2407–3066) and the N-terminal portions of ApxIIA (1–549) and ApxIVA C1, C2 (1–1500, and 1441–2700) were selected from the analysis. The *apxIA* gene was amplified from *A. pleuropneumoniae* serotype 5, while *apxIIA* and *apxIVA* were amplified from *A. pleuropneumoniae* serotype 2. The primers for *apxIA*, *apxIIA*, and *apxIVA* were designed using sequences available in GenBank (Supplementary Table 5). Amplified *apxIA Ct* (660 bp), *apxIIA Nt* (522 bp), *apxIVA Ct* (1500 bp) and *apxIVA C2* (1260 bp) were purified and cloned into the pET-300 vector for expression in *E.coli*.

Transformed *E. coli* cells were cultured in LB broth with ampicillin at 100 µg/ml to an absorbance of 0.6 nm (OD_0.6nm_), at which time isopropyl β-D-1-thiogalactopyranoside (IPTG, 1 mM) was added and the cells were cultured for an additional 4 h. The cells were harvested and re-suspended in lysis buffer (20 mM Tris-HCl, 500 mM sodium chloride, 8 M urea, and 50 mM imidazole, pH 7.0). Next, nickel-nitrilotriacetic acid (Ni-NTA) chelate affinity chromatography was performed according to the manufacturer’s instructions. The bound protein was eluted with elution buffer (20 mM Tris-HCl, 500 mM sodium chloride, 8 M urea, and 500 mM imidazole, pH 7.0). The purified recombinant protein was analyzed by sodium dodecyl sulfate-polyacrylamide gel electrophoresis (SDS-PAGE) and Western blot using an anti-histidine antibody (Jackson ImmunoResearch, USA) and an alkaline phosphatase kit (BioRad, USA) following the manufacturer’s protocol.

### Cell culture and treatments

After an immortalized porcine alveolar macrophage cell line (IPAMs, 3D4/31, ATCC CRL-2844) were cultured in Roswell Park Memorial Institute medium (RPMI) 1640 (Gibco, USA) containing 10% fetal bovine serum (FBS; Gibco, USA), penicillin (100 U/mL), and streptomycin (100 µg/mL), the cells were cultured for 12 h at a concentration of 1 × 10^6^ cells/well in 6-well culture plates containing 2% FBS in the same media to stabilize the cells. Additionally, polymyxin B was applied at 10 µg/ml for 30 min before treatment with stimulants, recombinant Apx toxins (10 µg/mL) and DPBS (negative control).

### RNA sequencing

After 24 h of incubation, total RNA was extracted from the cells using an RNeasy Mini Kit (Qiagen, Germany) according to the manufacturer’s instructions. For quality control, RNA purity and integrity were evaluated via denaturing gel electrophoresis, determining the OD 260/280 ratio, and analysis using an Agilent 2100 Bioanalyzer (Agilent Technologies, USA). The total RNA concentration was measured using Quant-IT RiboGreen (Invitrogen, USA). To determine the integrity of the total RNA, samples were evaluated by using TapeStation RNA ScreenTape system (Agilent Technologies, USA). Only high-quality RNA preparations, with an RIN greater than 7.0, were used for RNA library construction.

A library was prepared with 1 µg of total RNA for each sample using an Illumina TruSeq mRNA Sample Prep Kit (Illumina, USA). The first step in this workflow involves purifying the poly-A containing mRNA molecules using poly-T-attached magnetic beads. Following purification, the mRNA is fragmented into small pieces using divalent cations under elevated temperature. The cleaved RNA fragments are copied into first strand cDNA using SuperScript II reverse transcriptase (Invitrogen, USA) and random primers, followed by second strand cDNA synthesis using DNA Polymerase I and RNase H. These cDNA fragments then go through an end repair process, the addition of a single ‘A’ base, and subsequent ligation of the indexing adapters. The products are then purified and enriched via PCR to create the final cDNA library. The libraries were quantified using qPCR according to the qPCR Quantification Protocol Guide (KAPA Library Quantification Kits for Illumina Sequencing Platforms) and were qualitatively assessed using a TapeStation D1000 ScreenTape system (Agilent Technologies, USA). Indexed libraries were then sequenced using a HiSeq4000 platform (Illumina, USA) by Macrogen Incorporated.

### Read mapping and gene expression analyses

To estimate gene expression levels and to identify alternatively spliced transcripts, the RNA-Seq reads were mapped to the genome of *Sus scrofa* using TopHat^73^, which is capable of reporting split-read alignments across splice junctions, and evaluated using Cufflinks^74^ with the default options. The transcript counts at the isoform level were calculated, and the relative transcript abundances were reported as FPKM (Fragments Per Kilobase of exon per million fragments mapped) using Cufflinks. In addition, novel transcript and novel alternative splicing transcripts were identified per sample. These results were obtained using the Cufflinks reference annotation based transcript (RABT) assembly method, which allows for the discovery of reference transcripts and novel transcripts using the–g option.

Raw data were calculated as FPKM (Fragments Per Kilobase of exon model per million mapped fragments) of each transcript in each sample using Cufflinks. We excluded the transcripts with zeroed FPKM values that were more than the total observed in the samples. Next, we added 1 to the FPKM values of the filtered transcripts to facilitate a log2 transformation. Filtered data were transformed by logarithm and normalized using the quantile normalization method. For each transcript, we conducted an independent t-test between the treatment and the control. Each transcript was determined using an independent t-test.

### Screening and functional analysis of DEGs

DEGs were obtained by analyzing the mRNA transcripts of IPAMs treated with Apx toxins. Genes showing significantly altered expressions were subjected to gene set enrichment analysis using the PANTHER classification database (http://www.pantherdb.org). DEGs were categorized by biological processes and molecular functions using the PANTHER classification database and Fisher’s exact test, to detect coordinated changes in predefined sets of related genes.

### Biological system analysis

Data were analyzed through the use of IPA (QIAGEN Inc., https://www.qiagenbioinformatic s.com/products/ingenuitypathway-analysis)^75^. Differentially expressed genes with adjusted *p* values of less than 0.05 were uploaded into the IPA program. Each gene was mapped to its corresponding gene object in the Ingenuity Knowledge Base. A biological function analysis was performed using IPA to compare the DEGs associated with disease and disorders, molecular and cellular functions, and physiological system development and function in Apx toxin-treated IPAMs. A right-tailed Fisher’s exact test was used to calculate the *p*-value for each biological function. Canonical pathways from the IPA library of canonical pathways were investigated to identify major biological pathways associated with the Apx toxin treatment of IPAMs. The significance of the association between the data set and the canonical pathway was determined based on two parameters, including (1) the ratio of the number of genes from the dataset that mapped to the pathway to the total number of genes that mapped to the canonical pathway and (2) the *p*-value calculated using Fisher’s exact test to determine the probability that the association between the genes in the data set and the canonical pathway results from chance alone.

### Expression analysis of selected genes by real-time PCR

After treatment with the four recombinant proteins for 2, 12, 24, and 48 h, total RNA was extracted from the cells using an RNeasy Mini Kit (Qiagen, Germany) according to the manufacturer’s instructions. The expression of 22 genes was also studied by real-time PCR to verify the findings from the RNA-Seq data analysis or to acquire additional information about major immune responses (qRT-PCR, Supplementary Table 6). The qRT-PCR reactions were performed using 1 µL of cDNA, a Rotor-Gene SYBR Green PCR Kit (Qiagen, Germany) and a Rotor-Gene Q real-time PCR cycler (Qiagen, Germany). The cycling parameters were as follows: 95 °C for 3 min, followed by 45 cycles of 95 °C for 15 sec, 30 sec at 60 °C and 30 sec at 72 °C with fluorescence detected during the extension phase. The expression level was determined via the 2^−ΔΔCt^ method using glyceraldehyde-3-phosphate dehydrogenase (GAPDH) as a reference gene. The relative expression level was compared to that observed in control cells to determine the fold change in expression for each gene.

### Statistical analysis

Statistical significance of internalization was analyzed by Student’s t-test or repeated measures ANOVA using GraphPad Prism version 7.00 for Windows, GraphPad Software, La Jolla California USA, www.graphpad.com. Genes were considered differentially regulated when *p* was < 0.05. When differences were determined to be significant, fold changes with respect to the control condition were represented as follows: fold change = the mean ratio of gene expression in toxin-treated cells/the mean ratio of gene expression in DPBS-treated cells.

## Supplementary information

Supplementary Information

## Data Availability

Raw files and normalized datasets are available from the Gene Expression Omnibus (GEO) https://www.ncbi.nlm.nih.gov/geo under the accession number GSE116263.
