# Supplementary material for: Global Gene Networks in 3D4/31 Porcine Alveolar Macrophages Treated with Antigenic Epitopes of Actinobacillus pleuropneumoniae ApxIA, IIA, and IVA
Source: Sci Rep. 2019 Mar 27;9:5269. doi: 10.1038/s41598-019-41748-3 (PMC6437162; doi:10.1038/s41598-019-41748-3)
Supplement: Supplementary file 1 — Supplementary Information [file 41598_2019_41748_MOESM1_ESM.pdf]

Global Gene Networks in 3D4/31 Porcine Alveolar Macrophages Treated with Antigenic Epitopes of *Actinobacillus pleuropneumoniae* ApxIA, IIA, and IVA

Suji Kim, Myung Whan Oh, Woo Bin Park and Han Sang Yoo*

Supplementary Table S1. Top 40 canonical pathways of key DEGs in IPAMs treated with ApxIA Ct.

	Ingenuity Canonical Pathways	-log (p-value)	Ratio	z-score
**Metabolic pathways**	Superpathway of Cholesterol Biosynthesis	3.74	0.169	N/A
	Cholesterol Biosynthesis I	2.93	0.205	N/A
	Cholesterol Biosynthesis II (via 24,25-dihydrolanosterol)	2.93	0.205	N/A
	Cholesterol Biosynthesis III (via Desmosterol)	2.93	0.205	N/A
	Superpathway of Inositol Phosphate Compounds	2.91	0.105	N/A
**Signaling pathways**	Apoptosis Signaling	6.38	0.211	1.147
	Role of PKR in Interferon Induction and Antiviral Response	4.99	0.268	N/A
	Role of Osteoblasts, Osteoclasts and Chondrocytes in Rheumatoid Arthritis	4.99	0.13	N/A
	TWEAK Signaling	4.86	0.286	0
	TNFR1 Signaling	4.85	0.24	-0.302
	PTEN Signaling	4.41	0.157	1.606
	Glucocorticoid Receptor Signaling	4.32	0.11	N/A
	Death Receptor Signaling	4.3	0.172	1
	Aryl Hydrocarbon Receptor Signaling	4.18	0.143	-0.277
	LXR/RXR Activation	4.07	0.148	2.324
	Integrin Signaling	4.01	0.123	-0.655
	Tight Junction Signaling	3.82	0.132	N/A
	Axonal Guidance Signaling	3.8	0.0983	N/A
	Insulin Receptor Signaling	3.72	0.136	0.229
	Huntington's Disease Signaling	3.65	0.113	-0.832
	Hypoxia Signaling in the Cardiovascular System	3.64	0.173	0
	Chronic Myeloid Leukemia Signaling	3.61	0.151	N/A
	PI3K/AKT Signaling	3.5	0.138	-1.698
	Small Cell Lung Cancer Signaling	3.47	0.159	N/A
	Regulation of the Epithelial-Mesenchymal Transition Pathway	3.45	0.122	N/A
	Phagosome Maturation	3.41	0.129	N/A
	Induction of Apoptosis by HIV1	3.32	0.18	0.302
	Molecular Mechanisms of Cancer	3.26	0.097	N/A
	Sirtuin Signaling Pathway	3.21	0.102	0.378
	Endoplasmic Reticulum Stress Pathway	3.11	0.286	N/A
	Acute Myeloid Leukemia Signaling	3.08	0.146	-1.732
	Colorectal Cancer Metastasis Signaling	3.04	0.107	-0.408
	Cell Cycle: G1/S Checkpoint Regulation	3.02	0.167	0.905
	Hepatic Fibrosis / Hepatic Stellate Cell Activation	3.01	0.115	N/A
	Amyotrophic Lateral Sclerosis Signaling	3	0.133	N/A
	Glioblastoma Multiforme Signaling	2.99	0.12	-0.688
	Docosahexaenoic Acid (DHA) Signaling	2.91	0.172	N/A
	p53 Signaling	2.91	0.135	0
	CD40 Signaling	2.87	0.152	-0.905
	ErbB2-ErbB3 Signaling	2.85	0.159	-0.905

Supplementary Table S2. Top 40 canonical pathways of key DEGs in IPAMs treated with ApxIIA Nt.

	Ingenuity Canonical Pathways	-log (p-value)	Ratio	z-score
Metabolic pathways	Cholesterol Biosynthesis I	2.48	0.205	N/A
	Cholesterol Biosynthesis II (via 24,25-dihydrolanosterol)	2.48	0.205	N/A
	Cholesterol Biosynthesis III (via Desmosterol)	2.48	0.205	N/A
	Superpathway of Cholesterol Biosynthesis	2.11	0.145	N/A
	Chondroitin Sulfate Degradation (Metazoa)	1.82	0.217	N/A
	Dermatan Sulfate Degradation (Metazoa)	1.82	0.217	N/A
	Superpathway of Inositol Phosphate Compounds	1.71	0.101	N/A
Signaling pathways	Role of BRCA1 in DNA Damage Response	6.14	0.238	-1
	Sirtuin Signaling Pathway	5.07	0.133	0.87
	Hereditary Breast Cancer Signaling	5.04	0.171	N/A
	LPS/IL-1 Mediated Inhibition of RXR Function	4.07	0.137	0.277
	Cell Cycle: G1/S Checkpoint Regulation	2.96	0.182	0.632
	Adipogenesis pathway	2.88	0.141	N/A
	PDGF Signaling	2.85	0.156	0.258
	Insulin Receptor Signaling	2.82	0.136	0
	Unfolded protein response	2.56	0.182	N/A
	NRF2-mediated Oxidative Stress Response	2.39	0.119	0.302
	ATM Signaling	2.33	0.143	-2.714
	ErbB2-ErbB3 Signaling	2.3	0.159	-1.265
	Integrin Signaling	2.28	0.114	1.964
	Apoptosis Signaling	2.24	0.144	0.832
	p53 Signaling	2.23	0.135	-0.277
	Chronic Myeloid Leukemia Signaling	2.03	0.132	N/A
	Xenobiotic Metabolism Signaling	1.98	0.102	N/A
	Protein Ubiquitination Pathway	1.97	0.104	N/A
	Ceramide Signaling	1.97	0.134	0.577
	DNA Double-Strand Break Repair by Homologous Recombination	1.96	0.286	N/A
	IGF-1 Signaling	1.96	0.13	0
	Role of CHK Proteins in Cell Cycle Checkpoint Control	1.94	0.158	-0.378
	Mitochondrial Dysfunction	1.92	0.112	N/A
	iNOS Signaling	1.92	0.167	0.816
	Small Cell Lung Cancer Signaling	1.91	0.136	N/A
	HIPPO signaling	1.91	0.136	-1.134
	GDNF Family Ligand-Receptor Interactions	1.86	0.139	0.302
	Cyclins and Cell Cycle Regulation	1.82	0.138	-1.134
	Sumoylation Pathway	1.8	0.127	1.897
	Tumoricidal Function of Hepatic Natural Killer Cells	1.74	0.208	N/A
	Renal Cell Carcinoma Signaling	1.64	0.129	-1
	Regulation of the Epithelial-Mesenchymal Transition Pathway	1.63	0.106	N/A
	STAT3 Pathway	1.62	0.133	-0.632

Supplementary Table S3. Top 40 canonical pathways of key DEGs in IPAMs treated with ApxIVA C1.

	Ingenuity Canonical Pathways	-log (p-value)	Ratio	z-score
Metabolic pathways	Superpathway of Cholesterol Biosynthesis	5.43	0.145	N/A
	Cholesterol Biosynthesis I	4	0.179	N/A
	Cholesterol Biosynthesis II (via 24,25-dihydrolanosterol)	4	0.179	N/A
	Cholesterol Biosynthesis III (via Desmosterol)	4	0.179	N/A
	NAD Phosphorylation and Dephosphorylation	3.71	0.25	N/A
	Superpathway of Geranylgeranyldiphosphate Biosynthesis I (via Mevalonate)	3.39	0.171	N/A
	Mevalonate Pathway I	3.06	0.185	N/A
	Ketogenesis	2.85	0.222	N/A
	Pyridoxal 5'-phosphate Salvage Pathway	2.27	0.0933	N/A
Signaling pathways	Phospholipase C Signaling	5.03	0.0843	-2.065
	Molecular Mechanisms of Cancer	4.88	0.0697	N/A
	Protein Kinase A Signaling	4.69	0.068	-0.853
	Osteoarthritis Pathway	4.27	0.0826	-1.069
	Role of NFAT in Cardiac Hypertrophy	4.23	0.085	N/A
	Sirtuin Signaling Pathway	3.81	0.0679	0.5
	Actin Nucleation by ARP-WASP Complex	3.01	0.125	-0.447
	GNRH Signaling	2.92	0.08	-0.302
	Neuregulin Signaling	2.89	0.0968	-1.633
	Paxillin Signaling	2.85	0.0885	-1.667
	Integrin Signaling	2.8	0.0682	-1.941
	ILK Signaling	2.77	0.0704	-1.069
	Role of Macrophages, Fibroblasts and Endothelial Cells in Rheumatoid Arthritis	2.71	0.0597	N/A
	iNOS Signaling	2.65	0.125	0.447
	Oncostatin M Signaling	2.6	0.147	-0.447
	Hereditary Breast Cancer Signaling	2.52	0.0753	N/A
	Axonal Guidance Signaling	2.51	0.0524	N/A
	Calcium-induced T Lymphocyte Apoptosis	2.44	0.1	-0.447
	Agrin Interactions at Neuromuscular Junction	2.44	0.1	-1.89
	Telomerase Signaling	2.36	0.0811	-1
	Regulation of Actin-based Motility by Rho	2.34	0.087	-0.707
	Inhibition of Angiogenesis by TSP1	2.33	0.128	N/A
	PI3K Signaling in B Lymphocytes	2.29	0.0741	-1.897
	Adipogenesis pathway	2.29	0.0741	N/A
	Hypoxia Signaling in the Cardiovascular System	2.27	0.0933	-0.447
	Regulation of Cellular Mechanics by Calpain Protease	2.23	0.103	N/A
	α-Adrenergic Signaling	2.2	0.0825	-1.414
	PPARα/RXRα Activation	2.18	0.0649	0.333
	ErbB Signaling	2.17	0.0816	N/A
	Nur77 Signaling in T Lymphocytes	2.16	0.1	N/A
	p38 MAPK Signaling	2.14	0.075	1

Supplementary Table S4. Top 40 canonical pathways of key DEGs in IPAMs treated with ApxIVA C2.

	Ingenuity Canonical Pathways	-log (p-value)	Ratio	z-score
**Metabolic pathways**	Ketogenesis	2.33	0.222	N/A
	Superpathway of Cholesterol Biosynthesis	2.28	0.108	N/A
	Ketolysis	2.24	0.211	N/A
**Signaling pathways**	Osteoarthritis Pathway	6.3	0.119	-1.043
	Molecular Mechanisms of Cancer	4.23	0.0821	N/A
	Granulocyte Adhesion and Diapedesis	4.01	0.105	N/A
	TGF-β Signaling	3.83	0.138	-1.265
	LXR/RXR Activation	3.81	0.117	1.508
	Hepatic Fibrosis / Hepatic Stellate Cell Activation	3.7	0.0995	N/A
	VDR/RXR Activation	3.59	0.139	-0.447
	Actin Nucleation by ARP-WASP Complex	3.51	0.161	-1.134
	Agrin Interactions at Neuromuscular Junction	3.41	0.143	-1.414
	Sirtuin Signaling Pathway	3.31	0.0802	-0.853
	Agranulocyte Adhesion and Diapedesis	3.23	0.0938	N/A
	PI3K/AKT Signaling	3.22	0.108	-2.111
	Ephrin Receptor Signaling	3.17	0.0955	-0.302
	Integrin Signaling	2.94	0.0864	-0.471
	Axonal Guidance Signaling	2.89	0.0699	N/A
	p53 Signaling	2.85	0.108	-1.265
	Regulation of Cellular Mechanics by Calpain Protease	2.73	0.138	0
	IL-10 Signaling	2.66	0.123	N/A
	Leukocyte Extravasation Signaling	2.62	0.0829	-0.258
	RhoGDI Signaling	2.62	0.0874	0.905
	Glucocorticoid Receptor Signaling	2.59	0.0725	N/A
	PPARα/RXRα Activation	2.57	0.0865	0.632
	Huntington's Disease Signaling	2.55	0.0781	-0.378
	Toll-like Receptor Signaling	2.54	0.118	0.333
	Role of Osteoblasts, Osteoclasts and Chondrocytes in Rheumatoid Arthritis	2.53	0.0795	N/A
	MIF-mediated Glucocorticoid Regulation	2.53	0.162	0
	Antiproliferative Role of TOB in T Cell Signaling	2.51	0.192	N/A
	Virus Entry via Endocytic Pathways	2.46	0.102	N/A
	Tec Kinase Signaling	2.46	0.0867	0
	Neuregulin Signaling	2.45	0.108	-0.816
	IL-1 Signaling	2.38	0.105	-0.378
	Cell Cycle: G1/S Checkpoint Regulation	2.38	0.121	0
	Macropinocytosis Signaling	2.35	0.111	0
	G Beta Gamma Signaling	2.29	0.102	-1.897
	Unfolded protein response	2.26	0.127	N/A
	Signaling by Rho Family GTPases	2.24	0.0748	-0.277
	Xenobiotic Metabolism Signaling	2.21	0.0717	N/A

Supplementary Table S5. Nucleotide sequences of primers used in this study.

Region		Sequences	Size	Location
ApxIA Ct	F	CACCtttgagtgtcaatacaacgtattattagg	660	2407-3066
	R	agcagattgtgttaaataattactgaa		
ApxIIA Nt	F	CACCatgtcaaaaatcactttgtcatcat	522	1-525
	R	ttctaattgattaggatctttattttgaa		
ApxIVA C1	F	CACCatgacaaaattaactatgcaagatgtg	1500	1-1500
	R	actttttaactttttaacggcgggcaattttag		
ApxIVA C2	F	CACCactaattatcgttatgaagttaaaggac	1260	1441-2700
	R	gctatcggtgctttctagcg		

Supplementary Table S6. Validation of gene expression by RNA-Seq and quantitative real-time PCR.

Gene symbol	Accession number or Reference	Forward sequence (5'→3')	Reverse sequence (5'→3')
HMGCS1	NM_001252215.1	GGGTCACATTTGGTGCCTGGA	GCGTTCAAAGGAAGTGACCCA
MGP	NM_214116.1	CAGCAGAGATGGAGAGCGAA	CGCTGCCGGAAATAACGATT
ABCG1	XM_021071021.1	CAGTGTGTGTACCGGGGAAA	TGTTCTGATCGCCGTACTCG
NTS	XM_003481758.4	ATGGCTTTAGCTTGGAGGCA	GTTGAAAGGCCCTGCTGTGA
VGF	XM_021086257.1	CCTCCGTATCCTTGCTGCTT	AATGGGAAAACGCCTGCAAC
GM-CSF/CSF-2	Meurens et al. (2009)	GAAACCGTAGACGTCGTCTG	GTGCTGCTCATAGTGCTTGG
IL5/EDF	Meurens et al. (2009)	TGGAGCTGCCTACGTTAGTG	TCGCCTATCAGCAGAGTTCG
IL6/IFN beta 2	Meurens et al. (2009)	ATCAGGAGACCTGCTTGATG	TGGTGGCTTTGTCTGGATTC
IL12 p35	Meurens et al. (2009)	GGCCTGCTTACCACTTGAAC	GCATTCATGGCCTGGAACTC
STAT3	Meurens et al. (2009)	TGCAGCAGAAAGTGAGCTAC	CCGGTCTTGATGACTAATGG
STAT6	Meurens et al. (2009)	TCCCAGCTACGATCAAGATG	AGTGAGAGTGTGGTGGATAC
TGF beta	Meurens et al. (2009)	GAAGCGCATCGAGGCCATTC	GGCTCCGGTTCGACACTTTC
TNF alpha/TNFSF2	Meurens et al. (2009)	CCAATGGCAGAGTGGGTATG	TGAAGAGGACCTGGGAGTAG
PPAR gamma	Bassaganya-Riera et al. (2006)	AAGACGGGGTCCTCATCTCC	CGCCAGGTCGCTGTCATCT
CASP6	XM_013989349.2	CCTAATGCTTTGTATTTGTACGTCT	TTCAATTGGCTGACCCTCCAT
APAF1	XM_021093026.1	CGAGCCCTAATTGCTTTCGG	AAAAGCGAGGCGCAAACTTA
CYCS	NM_001129970.1	CAGAGCAAGATTCACCTGTGTT	TTGGCTCATGCCTTAACAGG
GAPDH	Sai T et al. (2011)	GCATCCTGGGCTACA	CTTTACTCCTTGGAGGCCATG
LMNA	AH014884.2	GCAACTCTAGACCCCGAACC	CGCAGGAGTGACTGTCTAGG
Bak	Xu YN et al. (2010)	CTAGAACCTAGCAGCACCAT	CGATCTTGGTGAAGTACTC
CASP9	Zhang et al. (2013)	GGCTGTCTACGGCACAGATGG	CTGGCTCGGGGTTACTGCCAG
CASP3	Xu YN et al. (2010)	GAGGCAGACTTCTTGTATGC	CATGGACACAATACATGGAA
Bax	Fan X et al. (2017)	GCCGAAATGTTTGCTGACG	CCGATCTCGAAGGAAGTCCA

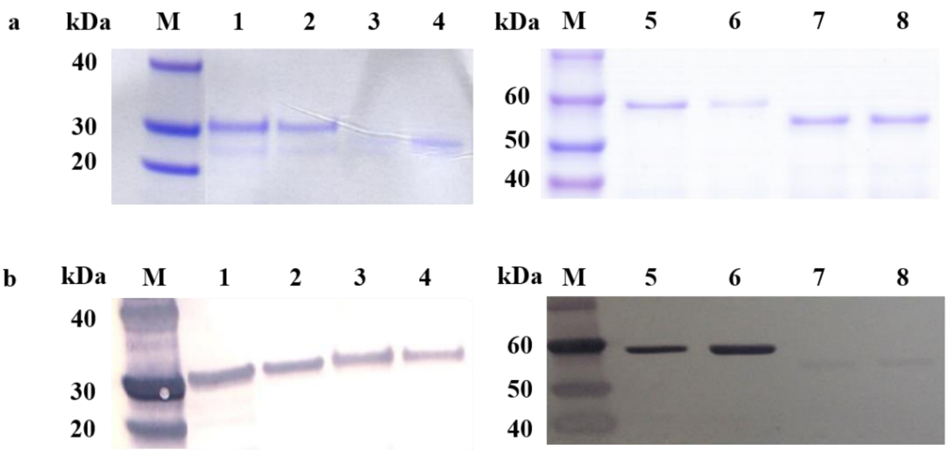

Supplementary Figure S1. Purification of the recombinant Apx toxins. (a) The recombinant ApxIA Ct, ApxIIA Nt, ApxIVA C1 and ApxIVA C2 epitopes were analyzed by SDS-PAGE and (b) Western blot with an anti-histidine antibody. Lane M: Protein standards (Novex Sharp Pre-stained Protein Standard; Life Technologies, USA); lanes 1 and 2: ApxIA Ct; lanes 3 and 4: ApxIIA Nt; lanes 5 and 6: ApxIVA C1; lanes 7 and 8: ApxIVA C2. Full-length blots/gels are presented in Supplementary Figure S1-1.

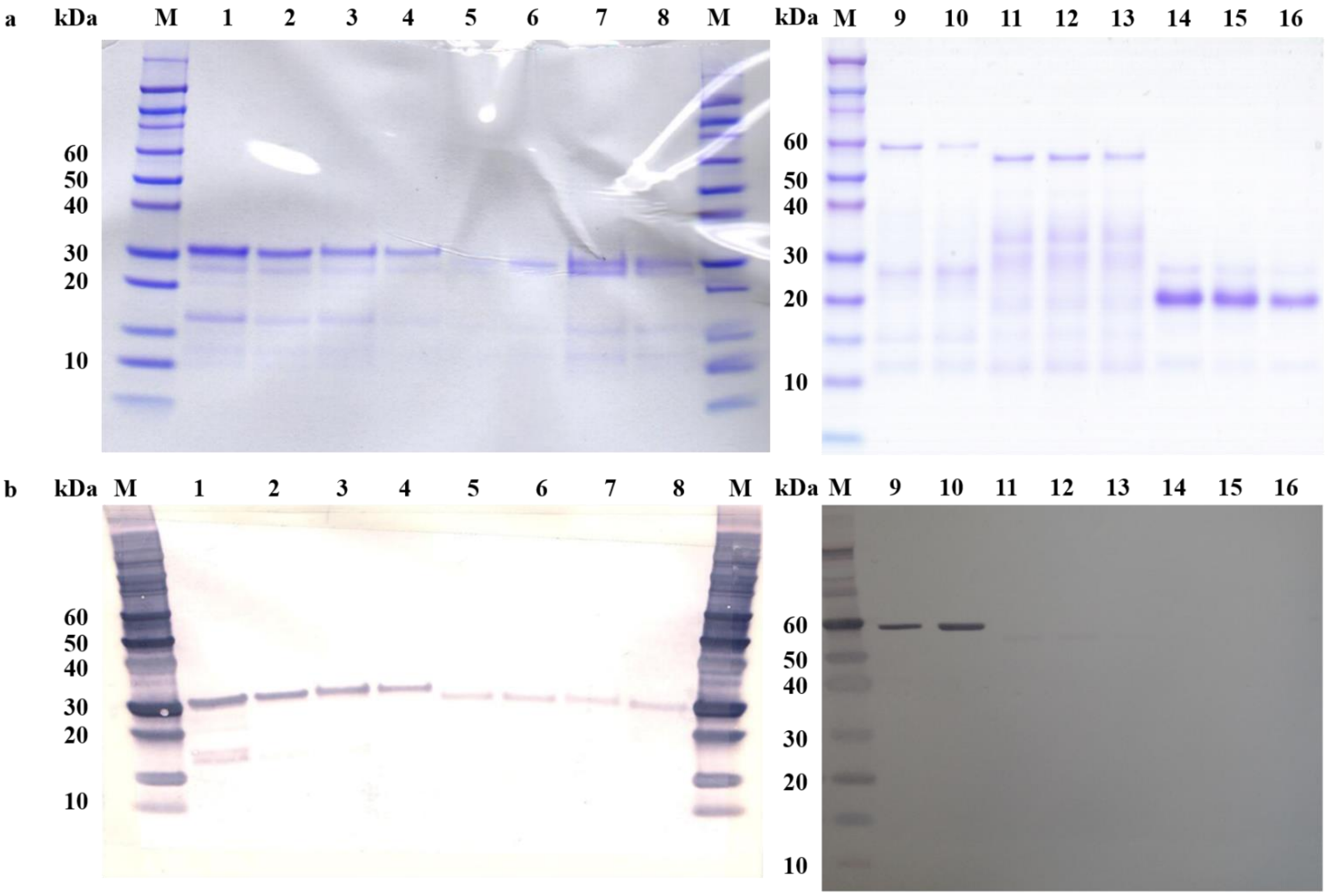

Supplementary Figure S1-1. Purification of the recombinant Apx toxins. (a) The recombinant ApxIA Ct, ApxIIA Nt, ApxIVA C1 and ApxIVA C2 epitopes were analyzed by SDS-PAGE and (b) Western blot with an anti-histidine antibody. Lane M: Protein standards (Novex Sharp Pre-stained Protein Standard; Life Technologies, USA); lanes 1-4: ApxIA Ct; lanes 5-8: ApxIIA Nt; lanes 9 and10: ApxIVA C1; lanes 11-13: ApxIVA C2; lanes 14-16: other protein.

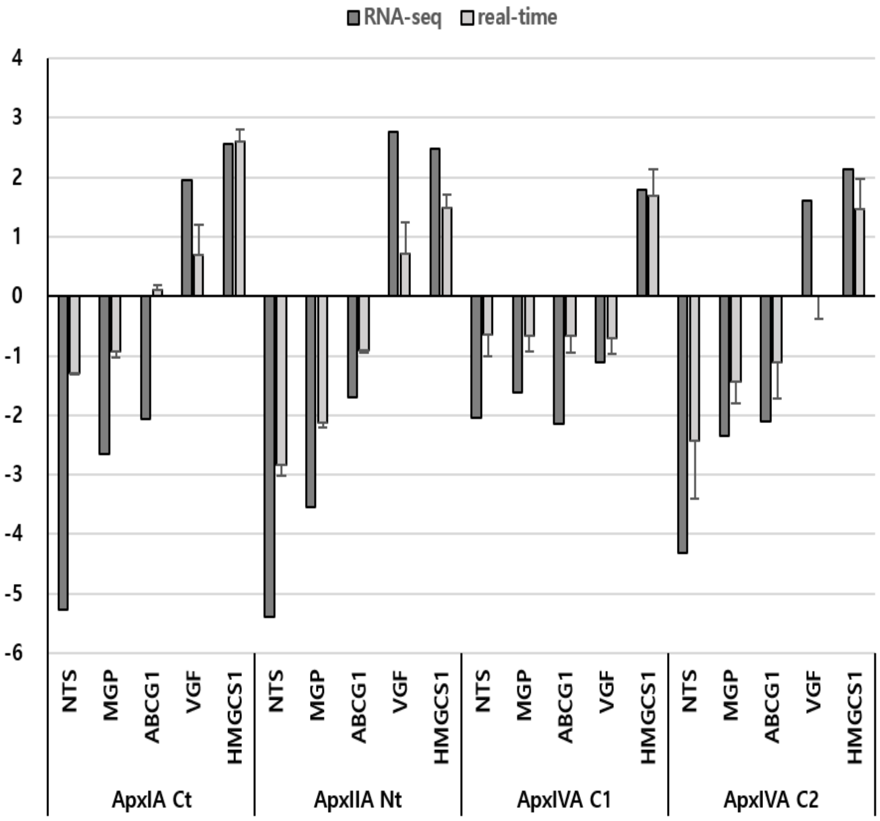

Supplementary Figure S2. Validation of gene expression by RNA-Seq and quantitative real-time PCR.

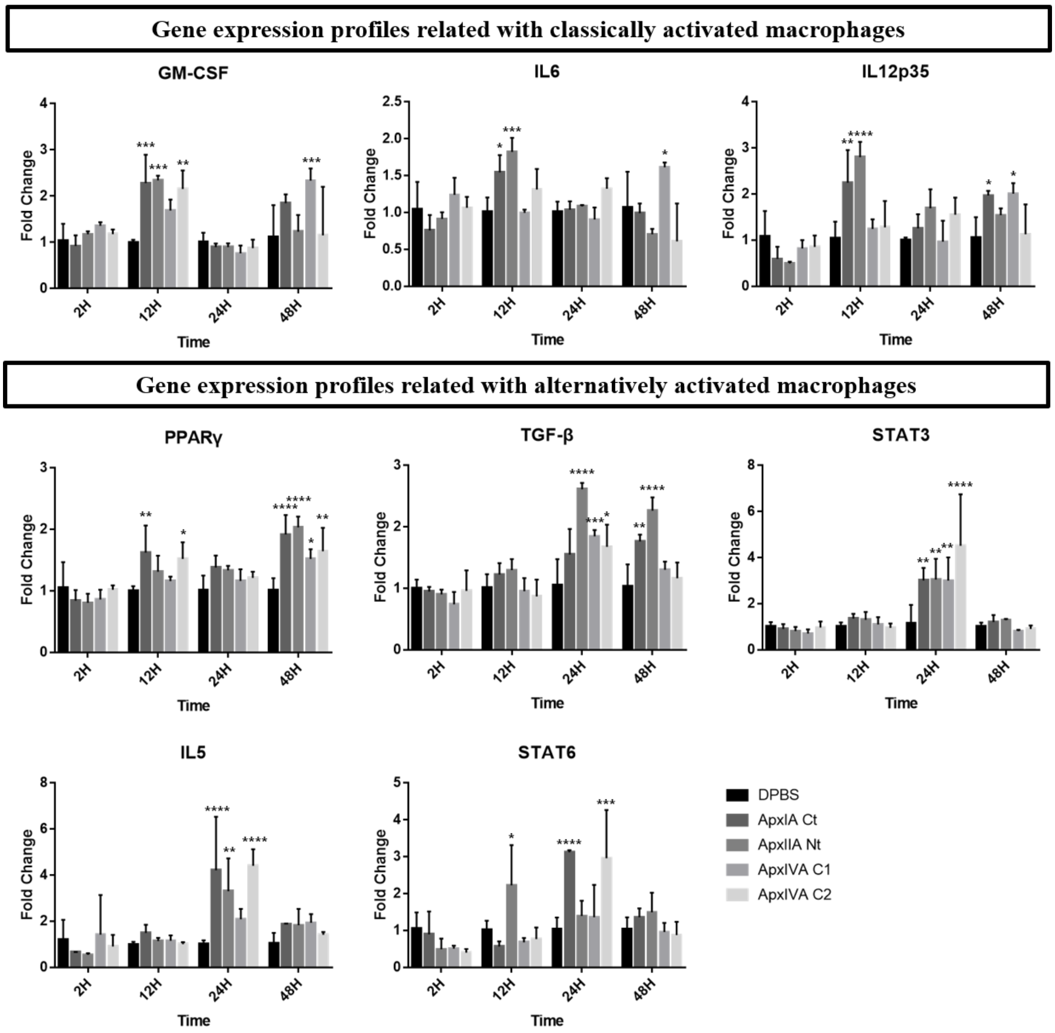

Supplementary Figure S3. Gene expression profiling associated with macrophage activation in the IPAMs-stimulated with Apx toxins.

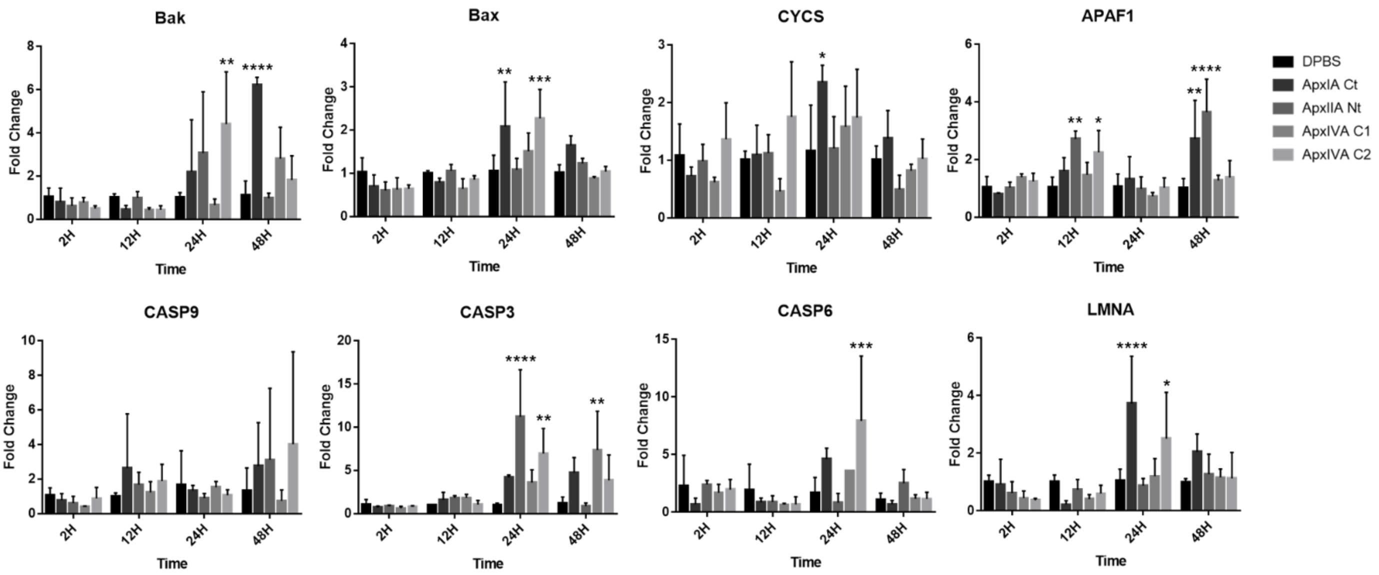

Supplementary Figure S4. Gene expression profiling associated with Apoptosis signaling in the IPAMs-stimulated with Apx toxins
